# Smart Lenses with Electrically Tuneable Astigmatism

**DOI:** 10.1038/s41598-019-52168-8

**Published:** 2019-11-06

**Authors:** Michele Ghilardi, Hugh Boys, Peter Török, James J. C. Busfield, Federico Carpi

**Affiliations:** 1 School of Engineering and Materials Science, Queen Mary University of London, London, UK; 2 Materials Research Institute, Queen Mary University of London, London, UK; 3 Department of Industrial Engineering, University of Florence, Florence, Italy; 4 School of Electronic Engineering and Computer Science, Queen Mary University of London, London, UK; 5 I+s.r.l, Florence, Italy; 6 University of Ulster, Belfast, UK; 7 School of Physical and Mathematical Sciences, NTU, Singapore, Singapore; 8 Department of Physics, Imperial College, London, UK

**Keywords:** Mechanical engineering, Materials for optics

## Abstract

The holy grail of reconfigurable optics for microscopy, machine vision and other imaging technologies is a compact, in-line, low cost, refractive device that could dynamically tune optical aberrations within a range of about 2–5 wavelengths. This paper presents the first electrically reconfigurable, fully elastomeric, tuneable optical lenses with motor-less electrical controllability of astigmatism in the visible range. By applying different voltage combinations to thin dielectric elastomer actuator segments surrounding a soft silicone lens, we show that the latter can be electrically deformed either radially or along selectable directions, so as to tune defocus or astigmatism, up to about 3 wavelengths. By mounting the new lenses on a commercial camera, we demonstrate their functionality, showing how electrically reconfiguring their shape can be used to dynamically control directional blurring while taking images of different targets, so as to emphasize directional features having orthogonal spatial orientations. Results suggest that the possibility of electrically controlling aberrations inherent to these smart lenses holds promise to develop highly versatile new components for adaptive optics.

## Introduction

Over the past couple of decades the field of adaptive optics has triggered a growing interest for optical lenses with tuneable focal length (varifocal lenses)^1^. They are desirable to reduce the complexity, size, weight, response time and costs of conventional focusing systems based on rigid lenses moved by motors. Several strategies have been explored, covering a range of physical principles and materials, including elastomeric or liquid lenses that are made tuneable either by deforming them mechanically with conventional motors^2–4^ or by using thermal driving^5^, liquid crystals^6–8^, electrowetting^9,10^, tuneable acoustic gradient index of refraction^11,12^, and dielectric elastomer actuation^13,14^. Some tuneable focal length lenses have also entered the market^15–17^.

Recently, there have been initial efforts to progress beyond such lenses with tuneable focal length, towards lenses capable of active control of optical aberrations other than just defocus^18–21^. Indeed, compact devices allowing for dynamic tuneability of optical aberrations could open up new opportunities to improve a diversity of existing technologies and devise entirely new ones. For instance, the ‘holy grail’ of reconfigurable optics for refractive imaging (i.e. microscopy, machine vision, etc.) is a low cost, compact, in-line refractive device with a tuning range of about 2–5 wavelengths (which is a practical range for specimen-induced aberrations^22,23^). However, approaches reported so far have been affected by the need for bulky actuators^18^, the use of optical media consisting of liquids susceptible to gravitational sagging^19^, operation outside the visible range^20^, or limited design versatility and/or tuning range^21,24^. So, at present the reference technology is still deformable mirrors and either reflective or transmissive spatial light modulators, used in astronomy and microscopy^25^. These devices have either limited tuning range or poor light efficiency, and usually are plagued by bulky design, complex electronics and high costs.

Here, we present the first electroactive, fully elastomeric (soft solid) lenses with tuneable astigmatism, working in the visible range. As a practical demonstration, we show how an electrical modulation of astigmatism of these new lenses mounted on conventional cameras can be used to dynamically control directional blurring while taking images, so as to emphasize directional features having different spatial orientation.

## Results

### Tuneable lens structure and fabrication

The proposed concept is based on special electromechanically active polymer (EAP) transducers^26^, known as dielectric elastomer actuators (DEAs)^27,28^. They represent a smart material technology for ‘artificial muscles’^29^. DEAs essentially are rubbery capacitors that can be significantly deformed by an applied voltage. In their simplest configuration, they consist of an insulating elastomeric membrane coated on either side with compliant electrode material. By applying an electric field across the soft dielectric layer, the generated electrical stress causes a reduction of its thickness and an expansion of its surface. This is the most effective approach to embed electrical actuation in a stretchable material, in order to electrically modulate its morphology. It offers large strains, fast response, low power consumption, low weight, small size, silent operation, ease of processing and versatility for design^28,29^. The DEA technology has been used to demonstrate tuneable lenses, consisting of transparent fluids enclosed between elastomeric membranes^13^. Using silicone membranes to actuate those structures has led to the world-fastest tuneable lenses made of smart materials, capable of response times down to ∼175 μs^14^. Moreover, that tuneable lens concept has also been demonstrated in a fully elastomeric (solid) version^30^.

**Figure 1:**
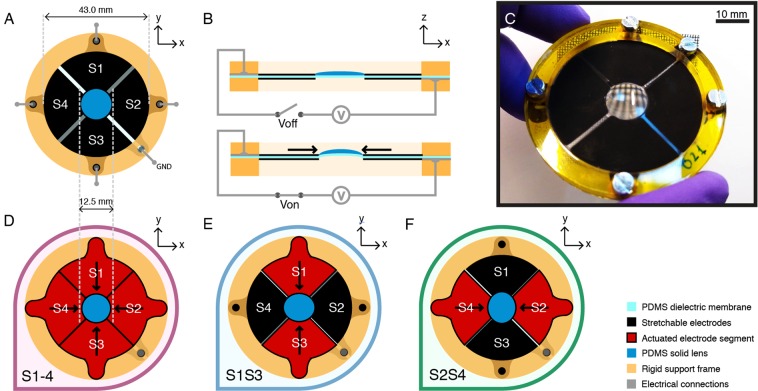
The smart lens and its possible operation modes. (**A**) Schematic view, showing the four DEA segments S1–S4 that surround the central elastomeric lens (ground electrode face down). (**B**) Schematic lateral sectional view, showing an electrical activation of the actuator segments that squeezes the lens, causing it to bend out of plane with a change of curvature, owing to its incompressibility. (**C**) Picture of a prototype lens: a grid pattern is seen magnified and distorted through the lens as the device is held tilted with respect to the grid’s plane. (**D**) A parallel activation of all four actuator segments causes the soft lens to undergo a radial uniform squeezing. (**E**)–(**F**) A parallel activation of opposite segments squeezes the lens in one direction.

Building on those achievements, the new device proposed here consists of a plano-convex soft solid-body lens, made of a silicone (polydimethylsiloxane – PDMS) elastomer, bonded to the inner circular region of an annular multi-segment DEA. One side of the actuator was patterned with an electrode divided into four independent segments (S1 to S4), which shared the same ground electrode on the other side of the device, as depicted in Fig. 1A.

This configuration allows for electrically controlling the lens astigmatism along two perpendicular axes (*x* and *y* in Fig. 1A). Indeed, by activating opposite electrode segments (e.g. S1 and S3), i.e. by applying the same voltage difference between each of them and the common ground electrode, the lens can be electrically squeezed along the direction of the activated segments. At the same time, the lens is stretched along the perpendicular direction, due to its incompressibility (Fig. 1E,F).

Furthermore, by activating the four segments S1-4 simultaneously (Fig. 1D), the resulting radial squeezing of the lens can be used to symmetrically increase its curvature and, thereby, reduce its focal length.

For any electrical activation pattern, the incompressible PDMS lens undergoes not only a change of curvature but also an out-of-plane bending, changing its shape from plano-convex to meniscus (Fig. 1B). This was verified experimentally by measuring the out-of-plane displacement of the lens’ surfaces upon actuation, using a laser sensor, as reported in the Supplementary Fig. S1.

**Figure 2:**
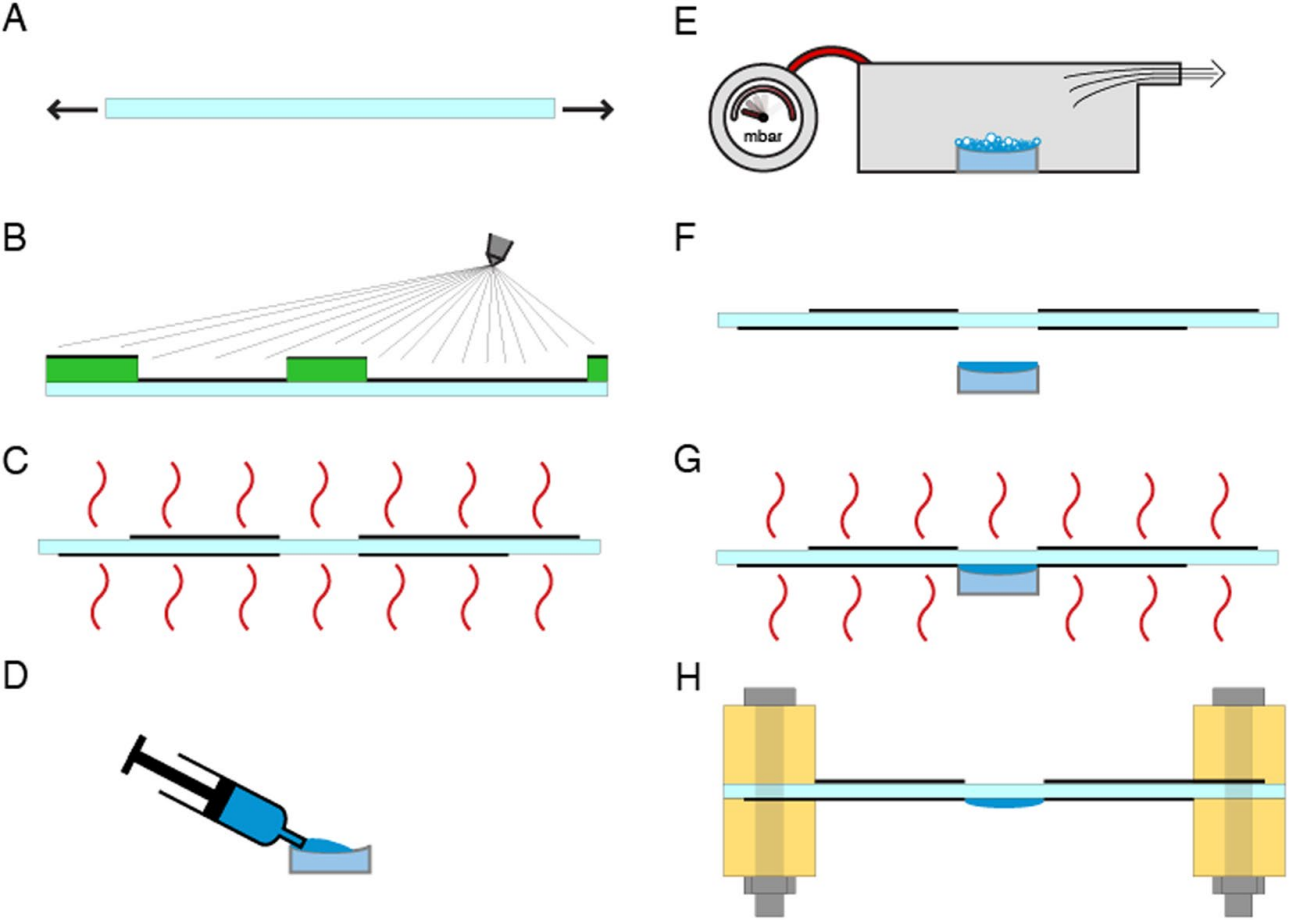
Manufacturing process of the smart lenses. (**A**) A PDMS membrane is stretched radially. (**B**) A mixture of liquid PDMS pre-polymer and carbon black is sprayed onto the membrane, using a mask to obtain the desired electrode pattern; the process is repeated on the other side of the membrane using a different mask. (**C**) The patterned electrode material is cross-linked in an oven, to complete the fabrication of the actuation part. (**D**) A liquid PDMS pre-polymer is poured in a concave glass lens used as a mold. (**E**) Air bubbles are removed in a vacuum chamber. (**F**) The patterned membrane is put in contact with the liquid PDMS pre-polymer in the mold, and they are aligned. (**G**) The device is put in an oven to cross-link the PDMS in the mold, obtaining a solid and stretchable lens irreversibly attached to the actuation membrane. (**H**) The lens is demolded and the device is completed with a rigid frame that supports the membrane via screws, which also act as electrical contacts.

The prototype lens shown in Fig. 1 was 12.5 mm wide, whilst its driving electrodes had an external diameter of 43 mm. The steps of its manufacturing process are depicted in Fig. 2.

PDMS was used for both the lens and the actuation membrane, because, as compared to alternatives like acrylic and polyurethane elastomers (which are also used in the DEA field), silicones have in general lower viscoelastic losses, allowing for higher stability over time and higher actuation speeds^14,31^. Details on the adopted materials are reported within the section Methods. The compliant electrodes were made of a silicone/carbon black composite. The soft lens was obtained by casting the PDMS within a commercial concave glass lens used as a mold, and cross-linking the material while it was maintained in contact with the pre-stretched actuation membrane, so as to obtain a monolithic structure (Fig. 2).

The resulting PDMS lens had a thickness of about 800 µm in the center, whilst that of the pre-stretched DEA membrane was approximately of 30 µm. The optical transmittances of the PDMS-based DEA membrane and of the lens’ constitutive PDMS was between 94.4% and 95.8% over the visible spectrum (Supplementary Fig. S2). The weight of the lens was 0.21 g (excluding the supporting frame, screws and electrical contacts, which could be designed and optimised in different ways, depending on the application needs).

The effect of the lens stiffness on the actuation performance of the DEA membrane is presented in the Supplementary Fig. S3. The extent of deformation imparted to the lens when at least one electrode segment is driven with a voltage *V* depends on the electrostatic pressure (Maxwell stress) *p* generated by the electric field *E* that builds up within the segment. That pressure can be estimated as^32^:

1 $$p={\varepsilon }_{0}{\varepsilon }_{r}{E}^{2}={\varepsilon }_{0}{\varepsilon }_{r}{(\frac{V}{d})}^{2}$$

where *ε*_0_ and *ε*_*r*_ are the dielectric permittivities of vacuum and the elastomer, respectively, and *d* is the dielectric membrane’s thickness. The electrically induced deformation of the lens determines its optical behaviour, whose characterization is presented below.

### Electrical tuneability of astigmatism and other optical aberrations

The lens aberrations were characterised in terms of defocus, astigmatism, coma and spherical aberration, via Zernike coefficients. In particular, with reference to Braat and Török^33^, we adopted the following notation for the Zernike expansion of the aberration function *f*(*ρ*,*ϑ*) in normalized cylindrical co-ordinates *ρ*,*ϑ* (0 ≤ *ρ* ≤ 1, with *ϑ* measured from the +*x* axis shown in Fig. 1):

2 $$f(\rho ,{\vartheta })=\sum _{n,m}{R}_{n}^{m}(\rho )[{a}_{n}^{m}\,\cos (m\vartheta )+{b}_{n}^{m}\,\sin (m{\vartheta })]$$

where ${R}_{n}^{m}$(*ρ*) is the radial Zernike polynomial of degree *n*, whilst ${a}_{2}^{0}$ and ${a}_{4}^{0}$ denote the Zernike coefficients for defocus (Defocus) and spherical aberration (SphA), ${a}_{2}^{2}$ and ${b}_{2}^{2}$ for horizontal and vertical astigmatism (Ast X and Ast Y), and ${a}_{3}^{1}$ and ${b}_{3}^{1}$ for horizontal and vertical coma (Coma X and Coma Y), respectively. We note that the Zernike polynomials are orthogonal within the unit circle.

**Figure 3:**
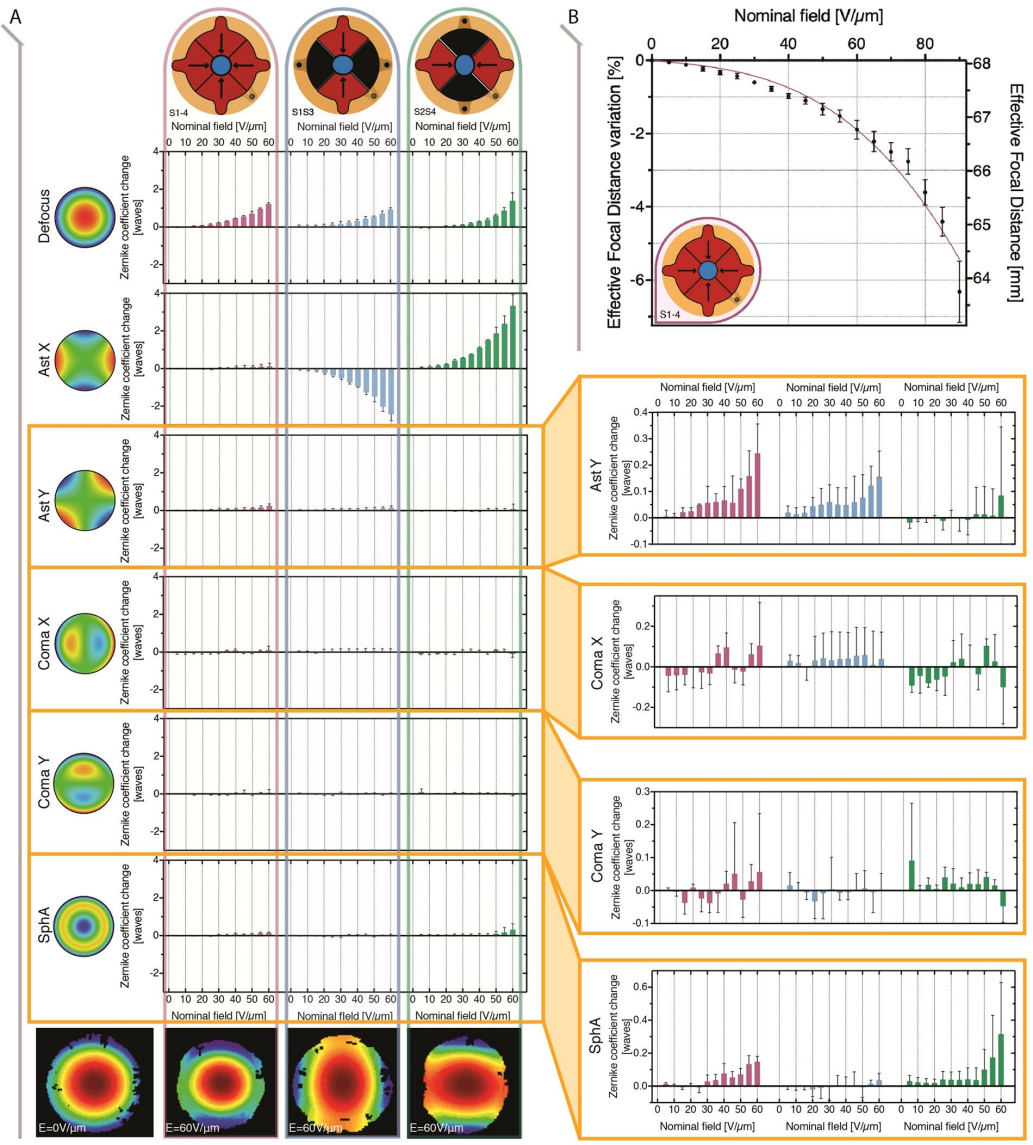
Electrical modulation of the smart lens aberrations and effective focal distance. (**A**) Each column corresponds to a different driving mode (combination of activated segments) and each row corresponds to a different aberration (Zernike coefficient). Each row-column intersection shows the dependence of the Zernike coefficient’s variation (with respect to its value at rest) on the applied electric field (nominal field, as the ratio between the applied voltage and the membrane’s thickness at electrical rest). Representing all the aberrations with the same scale (left-hand side of the figure) it is evident how defocus and Ast X have one-order-of-magnitude higher tuning ranges with respect to the others. The variations of Ast Y, coma and spherical aberration are zoomed in on the right-hand side. Error bars represent the standard deviation among 3 samples. The pictures at the bottom show the reconstructed wavefront at rest (left-hand side) and, for each driving mode, at the maximum applied field of 60 V/µm (for higher fields the lens deformation was such that the aperture seen by the interferometer changed from circular to elliptical, making the computation of the Zernike coefficients unreliable). (**B**) Variation of the effective focal distance (see definition within the text) on the applied electric field, for the smart lens driven in radial mode (S1–4), up to 90 V/µm.

These coefficients were assessed for each of the three driving modes (combinations of active segments) described in Fig. 1. By applying increasing voltages, we quantified the related variations of each coefficient with respect to its value when the lens is at electrical rest. Results are presented in Fig. 3A.

The electrical tuning range of defocus and horizontal astigmatism was found to be one-order-of-magnitude higher than those of the other Zernike coefficients. In particular, electrically squeezing the lens along one direction (S1S3 or S2S4) changed mostly defocus and horizontal astigmatism (Ast X) along the direction of actuation (*y* axis for S1S3 with Ast X decreasing upon actuation, and *x* axis for S2S4 with Ast X increasing). Among the other aberrations, Coma X and Coma Y did not show any significant variation. A more appreciable effect was evident for Ast Y and SphA (Fig. 3A). In particular, the increase of SphA when the lens was actuated along *x* (S2S4) was likely due to asymmetries introduced during the manufacturing process.

**Figure 4:**
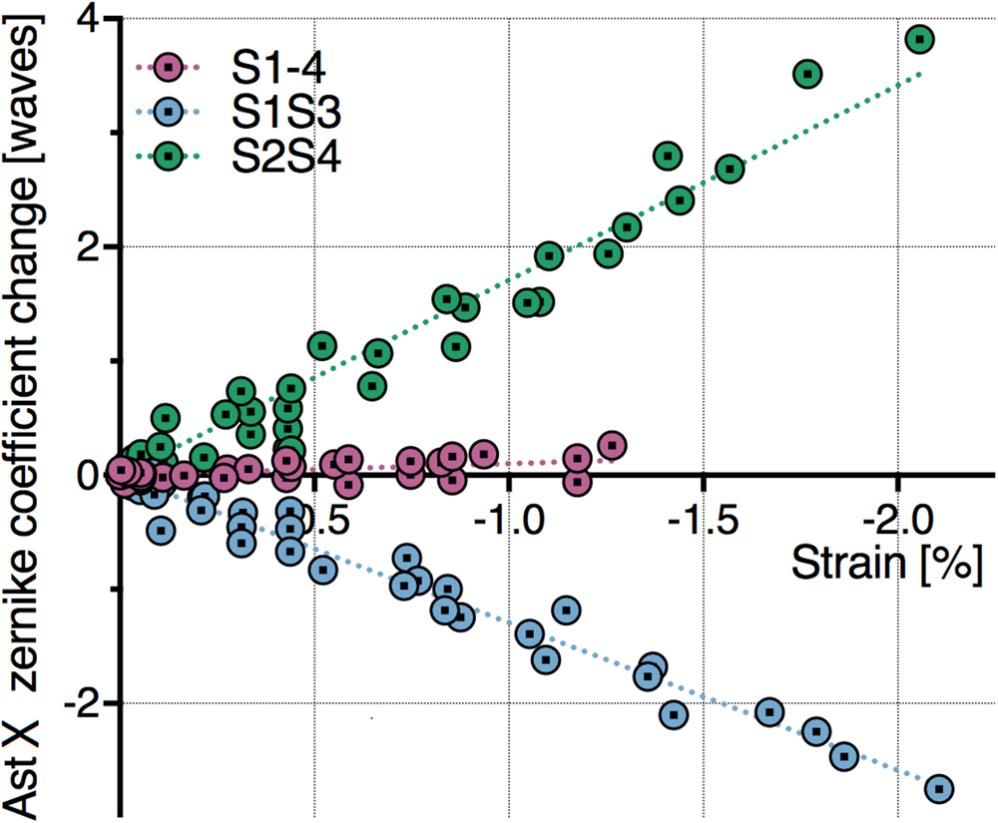
Dependence of the horizontal astigmatism (Ast X) of the smart lens on its electrically induced deformation, for the three driving modes. For each combination of active segments (S1S3, S2S4 and S1–4) and for increasing applied fields (up to 60 V/µm, with 5 V/µm steps), each average AstX value from the data set in Fig. 3A was matched with the corresponding average strain, at the same field, from the Supplementary Fig. S3, to obtain these AstX-strain plots (which therefore have no error bars). Linear fitting lines are added to each plot.

In order to show how each Zernike coefficient is affected by the electrically induced strain, for each operation mode, the Zernike coefficient-field data (Fig. 2) were matched with strain-field data (Supplementary Fig. S3). The results for horizontal astigmatism (Ast X) are presented in Fig. 4, whilst those related to the other aberrations are shown in the Supplementary Fig. S4.

Electrical deformations along one direction (S1S3 or S2S4) caused monotonic changes of the horizontal astigmatism, which depended on the active strain linearly (Fig. 4). In contrast, radial deformations (S1-4) did not have any significant effect on astigmatism (Fig. 4), as they primarily caused a change in defocus (Fig. 3A), due to the circular symmetry.

In addition to astigmatism and the other aberrations, the lens was also characterized in terms of focusing ability when it is driven with radial actuation (S1–4). To this end, we measured the Effective Focal Distance (EFD), here defined as the distance between the voltage-dependent focal point and the reference base plane at electrical rest (as detailed in Methods). It is worth noting that the EFD is slightly different from the focal length, as the position of the principal focal planes shifts while the lens is electrically deformed. Figure 3B presents the characterisation of the EFD, which decreased by 6.3% upon the application of an electric field of 90 V/µm. The setup used for the EFD measurement is shown in the Supplementary Fig. S5.

### Imaging with dynamically controllable astigmatism

A dynamically controllable astigmatism could be useful for imaging systems. As a test case, we investigated the possibility of combining the new lenses with conventional imaging technology, to selectively emphasize features that in a scene are aligned along different directions. To this end we performed the experiment described below.

**Figure 5:**
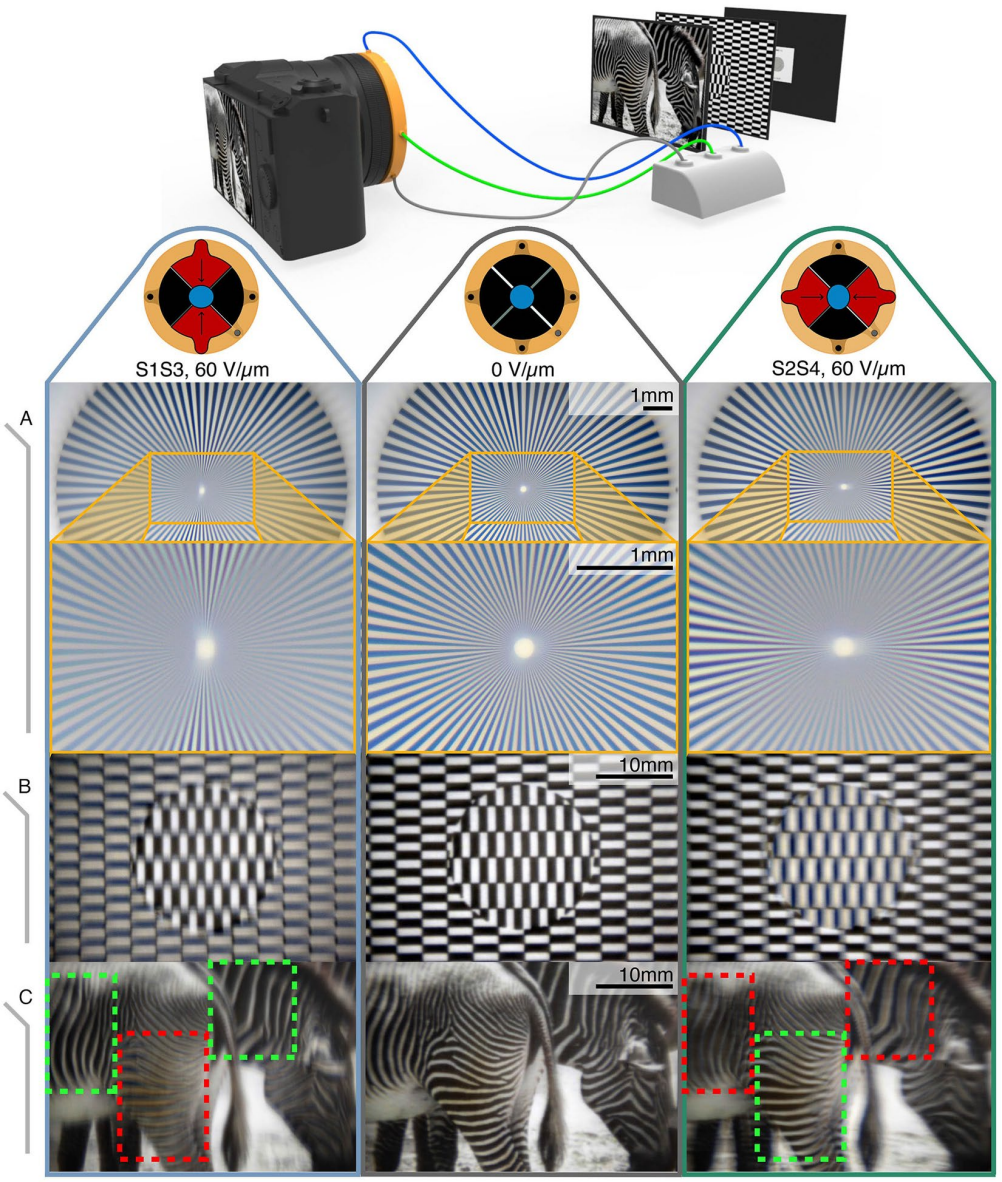
Demonstration of electrically controllable astigmatism for imaging with direction-dependent focusing. Pictures of different targets taken with a commercial camera and a smart lens mounted on it, without any extra lens (schematic drawing at the top). The lens was used in the three possible operation modes: electrical rest (central column) and with actuation along the vertical direction (left-hand column) or horizontal direction (right-hand column). The electrical activation of the segments S1S3 and S2S4 to take the left- and right-hand pictures, respectively, corresponded to an applied nominal electric field of 60 V/µm. The rows present images of the different selected targets: (**A**) A standard Siemens star target with 72 spokes, used to show astigmatism: when the lens was actuated along one direction, the image was blurred parallel to the direction of the lens squeezing; this directional blurring is more evident towards the center of the star target (zoomed in the second row), where the resolution necessary to resolve the spokes is higher. (**B**) A target with horizontal and vertical segments, used to show how electrically controlling the directional blurring can practically serve to selectively extract information along the two orthogonal directions. (**C**) Pictures of zebras, used to show the same effect applied to a non-geometrical pattern, so as to emphasize features having different spatial orientation in a real-life scene; in the central image all the zebra lines are in focus, whilst on the left- and right-hand pictures the vertical or horizontal (respectively) lines are more in focus (green boxes) than the orthogonal ones (red boxes).

A smart lens was mounted, using a custom-made adapter, onto a commercial reflex camera body (Supplementary Fig. S6), in front of the CMOS sensor, without any other optical component in the optical path. The camera with the smart lens was used to image three different targets having marked directional features (Fig. 5): a standard Siemens star target (R1L1S3P 72 bars, Thorlabs Inc., USA - conventionally used to estimate the resolution and the magnitude and orientation of aberrations in an optical system), a grid of vertical and horizontal segments, and a picture of zebras presenting areas of their coats with mainly vertically or horizontally aligned stripes.

The presence of astigmatism in an optical system implies that rays of the sagittal plane and rays of the meridional plane are focused at different planes perpendicularly to the optical axis. This effect results in a directional blurring of the gathered image. This is visible in Fig. 5, which presents the results of these tests. The central column shows images of the three targets taken through the smart lens at electrical rest, whilst the lateral pictures present the same target imaged while the lens was actuated along one direction (S1S3 or S2S4). The two pictures in Fig. 5B,C were obtained with a lens having an EFD at rest of 68 mm, whilst the image of Fig. 5A required a lens with a shorter EFD (45 mm), for a higher magnification (at the given fixed distance between lens and sensor) to resolve the thin spokes and so show the astigmatism introduced by the lens’ actuation.

In the Siemens star target, commonly used to test astigmatism, the spokes get thinner towards the center, thus needing a higher spatial resolution to be transmitted. When they are imaged through an astigmatic lens, the spokes parallel to the astigmatism’s direction are out of focus and unresolved. Moving away from the unresolved spokes along the circumferential direction, the other spokes get progressively less blurred, reaching maximum resolution perpendicularly to the astigmatism’s direction. Figure 5A shows how controlling astigmatism horizontally or vertically could reverse the directional blurring from one direction to the orthogonal one. This electrically tuneable directional blurring is also visible in Fig. 5B,C: by alternatively driving the two pairs of electrode segments, and so switching from one operation mode (S1S3) to the other (S2S4), it was possible to emphasize the vertical or horizontal features. The dynamic controllability is also shown in the Supplementary Videos SV1 and SV2.

## Discussion

The work presented here shows the feasibility and usable functionality of creating fully elastomeric varifocal lenses with electrically tuneable astigmatism in the visible range. The developed prototypes allowed for an electrical tuneability of astigmatism up to about 3 wavelengths (Fig. 3A), along two orthogonal directions.

Results of the imaging tests suggest a potential for straightforward applicability to existing imaging systems. In these tests, astigmatism was controlled to introduce temporary aberrations that could facilitate the extraction of directional features (Fig. 5). This was used as a simple example of dynamic processing of information at the optical level. From a practical standpoint, the most useful potential application in real systems could be for in-line dynamic correction of aberrations in optical imaging. For such uses, these new tuneable lenses would advantageously offer optical functionality and electromechanical actuation embedded within the same compact elastomeric structure, without moving parts or transmission mechanisms.

As a comparison, previous experimental works have described soft lenses with tuneable astigmatism using either external motors^18^, or acrylic-elastomer-based DEAs driving metalenses in the infrared range^20^. Differently, the new lenses described here combine visible-range operation, compact size, light weight, noise-free and energy efficient capacitive driving, as well as constitutive materials entirely based on PDMS silicones. The advantage of using silicones with respect to acrylic elastomers is in their typically lower viscoelastic losses, which ensure more stable performance and higher response speeds, with demonstrated ability of sub-millisecond operation^14^.

The developed manufacturing process, consisting of casting the PDMS lens in a concave glass lens used as a mold, is relatively simple and scalable. It can be implemented with low-cost materials and allows for optical designs with high versatility. Indeed, since any glass lens can be used as a mold with a highly polished controlled surface, it is possible to obtain PDMS lenses of high geometrical quality. The wide choice of available glass lenses straightforwardly translates into diversity not only of possible curvatures but also of shapes (such as aspheric lenses), to implement different optical behaviours. This widens the scope of applicability of these smart lenses as building blocks of adaptive optical systems. Molds fabricated with rapid prototyping techniques could become a viable alternative in the future, although controlling the surface properties (and so the optical quality) with comparable accuracy at the same cost might be a challenge.

We note that the Zernike aberrations produced by these deformable lenses are linearly not independent. However, this is not unusual for a diversity of adaptive optical devices. For example, deformable mirrors are known not to produce deformations that are orthogonal in terms of the Zernike polynomials. In order to obtain polynomials that are orthogonal over the surface of the deformable lens, a series of measurements should be carried out to determine the lens influence function that in turn can permit the control of the lens in an orthogonal manner.

We also note that, among the considered Zernike aberrations, this device configuration allowed us to control in a significant way only horizontal astigmatism and defocus (Fig. 3). In order to control all the aberration terms, it would be necessary to design different electrodes with suitable spatial configuration.

Therefore, whilst this work showed that it is possible to induce axially asymmetric aberrations, further studies are necessary to address the challenging development of lenses that allow for controlling all the aberrations, and each one independently from the others.

Additional future improvements of these deformable lenses could include an increase of the number of electrode segments, so as to tune astigmatism along more directions.

Moreover, the tuning range could be extended by increasing the electrically induced deformation. This could be achieved in two ways. i) using a softer PDMS for the lens, so that it can be compressed more by any given force exerted by the DEA; ii) creating a multi-layered active membrane, by stacking multiple dielectric films intertwined to multiple compliant electrodes; this would increase the membrane’s total thickness and so also the force acting on the lens, while preserving the same separation between electrode pairs and so avoiding the need for higher driving voltages.

However, the voltages required by the smart lenses shown in this work are nevertheless high. Indeed, the electric field of 60 V/µm was reached across a DEA membrane having a pre-stretched thickness of about 30 µm (see Methods), thus requiring an applied voltage of about 1.8 kV. The generation of voltages of that order of magnitude is not particularly problematic from a technical standpoint, or particularly dangerous in terms of electrical safety, considering that this technology does not need high driving powers, as the actuator behaves like a capacitive electrical load. Rather, the main drawback is introduced at the level of the size (and cost) of the required electronics. In this work, high voltage multipliers with a volume of about 2 cm^3^ were used (see Methods). They can generate up to 5 kV at 0.5 W from an input signal up to 5 V, being therefore suitable for battery-operated portable electronics. Such voltage multipliers make the overall system still competitively more compact than those possible with alternative technologies that modulate the astigmatism of soft lenses via external motors. Nevertheless, a further miniaturisation would be desirable for integrated systems. Downsizing the driving electronics from cube centimetres to cube millimetres requires lowering the driving voltages to a few hundred Volts. The targeted threshold is around 250 V, which is a typical driving voltage for the low-size (and low-cost) electronics of piezoelectric transducers available in a diverse range of products today. To reach that goal, according to Eq. (1) there are two strategies: (i) synthesis of new elastomers with a higher dielectric constant; (ii) usage of thinner actuation membranes. The first route is being explored in the DEA field with various approaches^28,34,35^. The second route requires improved manufacturing processes to reduce the thickness ideally down to a few microns. Although this is challenging for highly stretchable materials, the feasibility has been demonstrated^36^. On the other hand, in order to avoid a reduction of the force due to the membrane’s lower thickness, it would be necessary to create a multi-layer structure, as discussed above.

So, bespoke manufacturing processes are expected to play a key role in the future of this soft matter technology, to unleash its full potential. They are envisaged to benefit from research efforts on 3D printing techniques that are currently spent (separately, at present) for DEAs^37,38^, and high quality optical components^39^. Integrating these independent technologies could potentially enable the manufacture of cheap monolithic 3D optical devices with soft structure and electrically reconfigurable complex shapes.

## Methods

### Dielectric elastomer membrane

The elastomer used for the DEAs is a 50 µm-thick PDMS membrane (Elastosil 2030 250/50, Wacker, Germany), which was equi-biaxially prestretched by 1.3 times (30% prestrain), resulting in an estimated un-actuated thickness of 29.6 µm.

### Compliant electrode manufacturing

The compliant electrodes were manufactured by mask-spraying with an airbrush a PDMS-carbon black composite ink. The ink was obtained by mixing in a high speed planetary mixer (ARE250, Thinky, USA) a PDMS silicone pre-polymer (MED4910, Nusil, USA) with 9 wt% of carbon black (Black Pearls 2000, Cabot, USA) and solvents (isopropanol, isooctane). The sprayed mixture was cross-linked on the elastomer membrane in an oven at 80 °C for 45 minutes.

### Compact low-power electronics

The lenses were electrically driven with a custom-made multichannel high voltage system, using miniature high voltage multipliers (Q50, EMCO High Voltage Corporation, USA). The system was controlled by an Arduino board (Arduino micro, Arduino, Italy), interfaced to a laptop via serial communication.

### PDMS lens manufacturing

The lenses were made of a commercial silicone elastomer (Sylgard 184, Dow Corning, USA). The crosslinker-to-pre-polymer weight ratio was 1:20, lower than that recommended by the producer (1:10), so as to obtain softer lenses, more easily deformable by the DEA, allowing for a larger tuning range. Off-the-shelf glass concave lenses (LC1439 lens, Thorlabs, USA) having a radius of curvature of −25.7 mm and a diameter of 12.7 mm were used as molds. The PDMS pre-polymer mixed to the curing agent was poured within the glass lens and air bubbles were removed in a vacuum chamber. The elastomer was cross-linked within the glass lens (and in contact with the actuation membrane) in an oven at 80 °C for 1 hour.

### Active strain measurement

The active strain (along the *x* and *y* directions – see Fig. 1) of the circular region hosting the lens was measured for different voltage steps, corresponding to nominal electric field increments of 5 V/µm, up to 80 V/µm. The measurements were performed by taking pictures in response to each voltage step, using a reflex camera (EOS 1300D, Canon, Japan), which was triggered via a Java program. A light source on the opposite side of the DEA was used to increase the image contrast. The acquired pictures were processed in Matlab with a custom image processing script.

### Aberrations measurements

The measurements of the wavefront aberrations were carried out using an interferometer (GPI XP, Zygo, USA) examining the distortion of the transmitted wavefront at λ = 632.8 nm. The lens was placed in the collimated measurement beam, behind an 8 mm diaphragm aperture. A concave reference sphere mirror was used to reflect the transmitted wavefront back through the lens and into the interferometer, to create the interference fringe pattern. The fringe pattern produced by the interference among the reflected, distorted and transmitted beams was analysed with a commercial software (MetroPro, Zygo, USA), to extrapolate the Zernike coefficients representing the wavefront aberrations.

### Effective focal distance measurement

The setup used to determine the EFD is shown in the Supplementary Fig. S5. It comprised a laser source (TLS001-635, Thorlabs Inc., USA) and a beam expander setup. The collimated laser beam went through the tuneable lens and converged to the lens’ focal point. A 100 µm pinhole (P100S, Thorlabs Inc., USA) was placed in front of a photodetector (PDA100A EC, Thorlabs Inc., USA) and both of them were arranged on a translation stage that could be moved along the lens optical axis. By moving the stage back and forth, the pinhole’s distance from the lens corresponding to the highest value measured by the photodetector was recorded as the lens EFD.

## Supplementary information

Supplementary information

Supplementary video SV1

Supplementary video SV2

## References

[1] Zappe, H. & Duppé, C. *Tunable Micro-optics* (Cambridge University Press, 2015).

[2] Beadie G (2008). Tunable polymer lens. Opt. Express.

[3] Liebetraut P, Petsch S, Mönch W, Zappe H (2011). Tunable solid-body elastomer lenses with electromagnetic actuation. Appl. Opt..

[4] Marks R, Mathine DL, Peyman G, Schwiegerling J, Peyghambarian N (2010). Adjustable adaptive compact fluidic phoropter with no mechanical translation of lenses. Opt. Lett..

[5] Lee S, Tung H, Chen W, Fang W (2006). Thermal actuated solid tunable lens. IEEE Photonics Technol. Lett..

[6] Wang B, Ye M, Sato S (2004). Lens of electrically controllable focal length made by a glass lens and liquid-crystal layers. Appl. Opt..

[7] Lin H-C, Lin Y-H (2010). An electrically tunable focusing pico-projector adopting a liquid crystal lens. Jpn. J. Appl. Phys..

[8] Lin H-C, Lin Y-H (2010). A fast response and large electrically tunable-focusing imaging system based on switching of two modes of a liquid crystal lens. Appl. Phys. Lett..

[9] Ren H, Wu S-T (2007). Variable-focus liquid lens. Opt. Express.

[10] Berge B, Peseux J (2000). Variable focal lens controlled by an external voltage: An application of electrowetting. Eur. Phys. J. E.

[11] Mermillod-Blondin A, McLeod E, Arnold CB (2008). High-speed varifocal imaging with a tunable acoustic gradient index of refraction lens. Opt. Lett..

[12] Duocastella M, Sun B, Arnold CB (2012). Simultaneous imaging of multiple focal planes for three-dimensional microscopy using ultra-high-speed adaptive optics. J. Biomed. Opt..

[13] Carpi F, Frediani G, Turco S, De Rossi D (2011). Bioinspired tunable lens with muscle-like electroactive elastomers. Adv. Funct. Mater..

[14] Maffli L, Rosset S, Ghilardi M, Carpi F, Shea H (2015). Ultrafast all-polymer electrically tunable silicone lenses. Adv. Funct. Mater..

[15] Optotune. Available at, http://www.optotune.com/.

[16] Varioptic - Liquid lens solutions, Auto focus M12 and C-mount lens modules. Available at, http://www.varioptic.com/.

[17] Holochip. *holochip* Available at, https://www.holochip.com.

[18] Liebetraut P, Petsch S, Liebeskind J, Zappe H (2013). Elastomeric lenses with tunable astigmatism. Light Sci. Appl..

[19] Kopp D, Zappe H (2016). Tubular astigmatism-tunable fluidic lens. Opt. Lett..

[20] She A, Zhang S, Shian S, Clarke DR, Capasso F (2018). Adaptive metalenses with simultaneous electrical control of focal length, astigmatism, and shift. Sci. Adv..

[21] Adaptive Optics Technologies Company|Dynamic Optics. Available at, https://dynamic-optics.eu.

[22] Török P, Varga P, Laczik Z, Booker GR (1995). Electromagnetic diffraction of light focused through a planar interface between materials of mismatched refractive indices: an integral representation. JOSA A.

[23] Schwertner M, Booth MJ, Wilson T (2004). Characterizing specimen induced aberrations for high NA adaptive optical microscopy. Opt. Express.

[24] Lee S, Chen W, Tung H, Fang W (2007). Microlens with tunable astigmatism. IEEE Photonics Technol. Lett..

[25] Maurer C, Jesacher A, Bernet S, Ritsch-Marte M (2011). What spatial light modulators can do for optical microscopy. Laser Photonics Rev..

[26] Carpi, F. (ed.) *Electromechanically Active Polymers: A Concise Reference*. (Springer International Publishing, 2016).

[27] Pelrine R, Kornbluh R, Pei Q, Joseph J (2000). High-Speed Electrically Actuated Elastomers with Strain Greater Than 100%. Science.

[28] Carpi F., De Rossi D., Kornbluh R., Pelrine R., Sommer-Larsen P. (ed.) *Dielectric Elastomers As Electromechanical Transducers*. *Fundamentals*, *materials*, *devices*, *models & applications of an emerging electroactive polymer technology*. (Elsevier, 2008).

[29] Carpi F, Bauer S, De Rossi D (2010). Stretching dielectric elastomer performance. Science.

[30] Pieroni M, Lagomarsini C, De Rossi D, Carpi F (2016). Electrically tunable soft solid lens inspired by reptile and bird accommodation. Bioinspir. Biomim..

[31] Rosset S, Shea HR (2016). Small, fast, and tough: shrinking down integrated elastomer transducers. Appl. Phys. Rev..

[32] Pelrine RE, Kornbluh RD, Joseph JP (1998). Electrostriction of polymer dielectrics with compliant electrodes as a means of actuation. Sens. Actuators Phys..

[33] Braat, J. & Török, P. *Imaging Optics*. (Cambridge University Press, 2019).

[34] Madsen FB, Daugaard AE, Hvilsted S, Skov AL (2016). The current state of silicone-based dielectric elastomer transducers. Macromol. Rapid Commun..

[35] Dünki SJ, Ko YS, Nüesch FA, Opris DM (2015). Self-Repairable, High permittivity dielectric elastomers with large actuation strains at low electric fields. Adv. Funct. Mater..

[36] Poulin A, Rosset S, Shea HR (2015). Printing low-voltage dielectric elastomer actuators. Appl. Phys. Lett..

[37] Rossiter, J., Walters, P. & Stoimenov, B. Printing 3D dielectric elastomer actuators for soft robotics. In *Electroactive Polymer Actuators and Devices (EAPAD) 2009* **7287**, 72870H (SPIE, 2009).

[38] McCoul D, Rosset S, Schlatter S, Shea H (2017). Inkjet 3D printing of UV and thermal cure silicone elastomers for dielectric elastomer actuators. Smart Mater. Struct..

[39] Vaidya N, Solgaard O (2018). 3D printed optics with nanometer scale surface roughness. Microsyst. Nanoeng..

