# Supplementary material for: Smart Lenses with Electrically Tuneable Astigmatism
Source: Sci Rep. 2019 Nov 6;9:16127. doi: 10.1038/s41598-019-52168-8 (PMC6834852; doi:10.1038/s41598-019-52168-8)
Supplement: Supplementary file 1 — Supplementary information [file 41598_2019_52168_MOESM1_ESM.pdf]

Smart Lenses With Electrically Tuneable Astigmatism

Michele Ghilardi[1,2,3], Hugh Boys[4,5,6], Peter Török[7,8], James J.C. Busfield[1,2], Federico Carpi[3]

Supplementary Information

Video SV1

Video SV2

Supplementary videos. They show the geometrical target (video SV1) and the zebra target (video SV2) as seen through the smart lens while it was controlled so as to continuously change the astigmatism. The two electrode pairs of the lens were driven with sinusoidal signals having a phase delay of π, at a maximum nominal field of 60 V/μm and a frequency of 0.3 Hz. The lens gradually switched from one operation mode (S1S3) to the other (S2S4) and back, resulting in a transition of directional blurring of the image from vertical to horizontal. Each video shows a full actuation cycle.

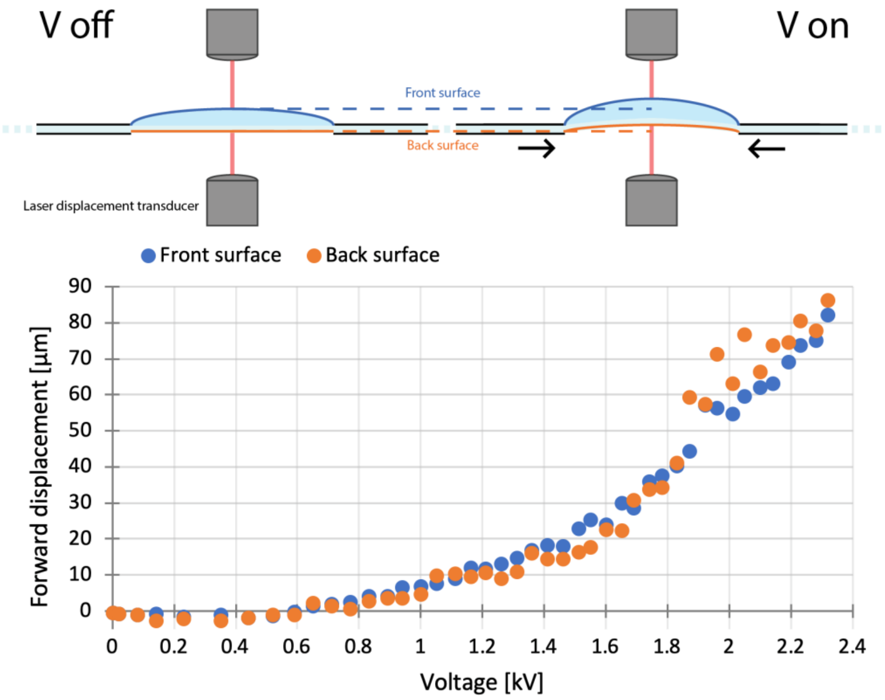

Figure S1. Out-of-plane displacement of the lens' front and back surfaces. To experimentally verify that the incompressible PDMS lens bends out-of-plane when radially squeezed by the surrounding DEA, the forward displacement of the central point of the front and back surfaces of the lens was measured using a laser displacement transducer. (Top) Schematic drawing of the setup. (Bottom) Measurement results. In order for the laser to be reflected on the transparent lens –and thus for the measurement to be carried out– a thin layer of PVA powder was sprinkled on the lens' surface (and then washed away with deionized water). The influence of the powder's grains diameter on the measurement was not such to invalidate the aim of this test, i.e. verifying the occurrence of an actual bending.

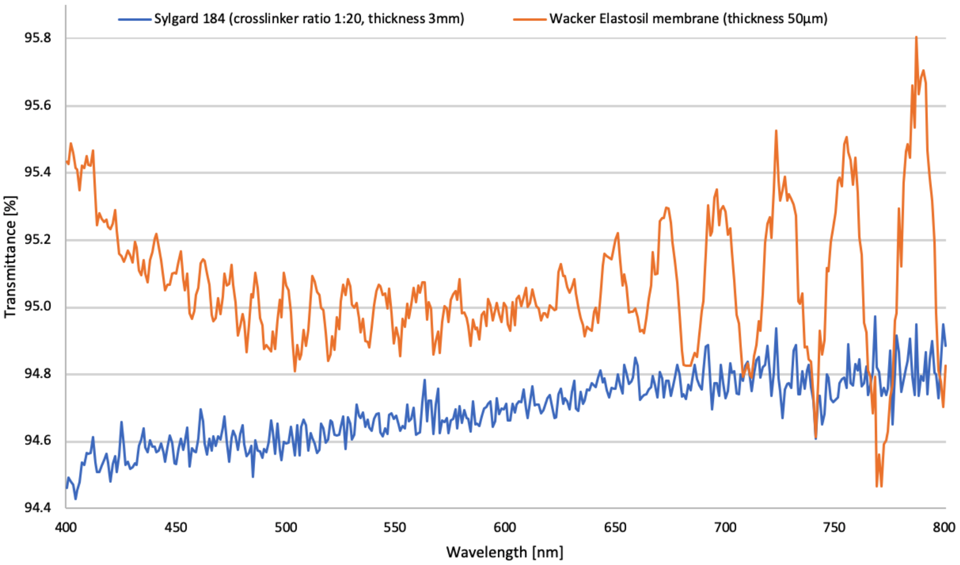

Figure S2. Transmittance of the employed PDMS elastomers. A UV-VIS machine was used to measure, over the visible spectrum, the transmittance of a sheet made of the PDMS that formed the lens (Sylgard 184, crosslinker ratio 1:20, thickness of 3 mm) and of the PDMS membrane adopted as the dielectric elastomer for the DEA (Wacker Elastosil 2030 250/50, thickness of 50µm).

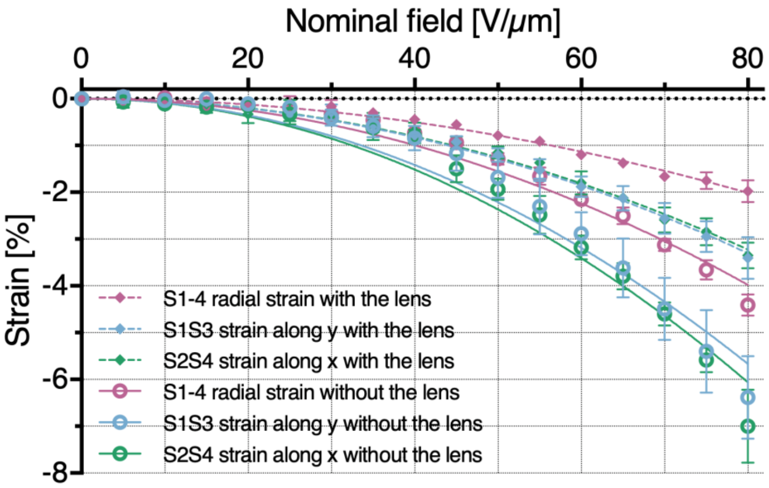

Figure S3. Active strain as a function of the applied electric field. The strain of the device is compared when the membrane is coated or not with the lens, to show the introduced stiffening. Error bars represent the standard deviation among 6 samples. Quadratic fitting lines are used as a guide to the eye.

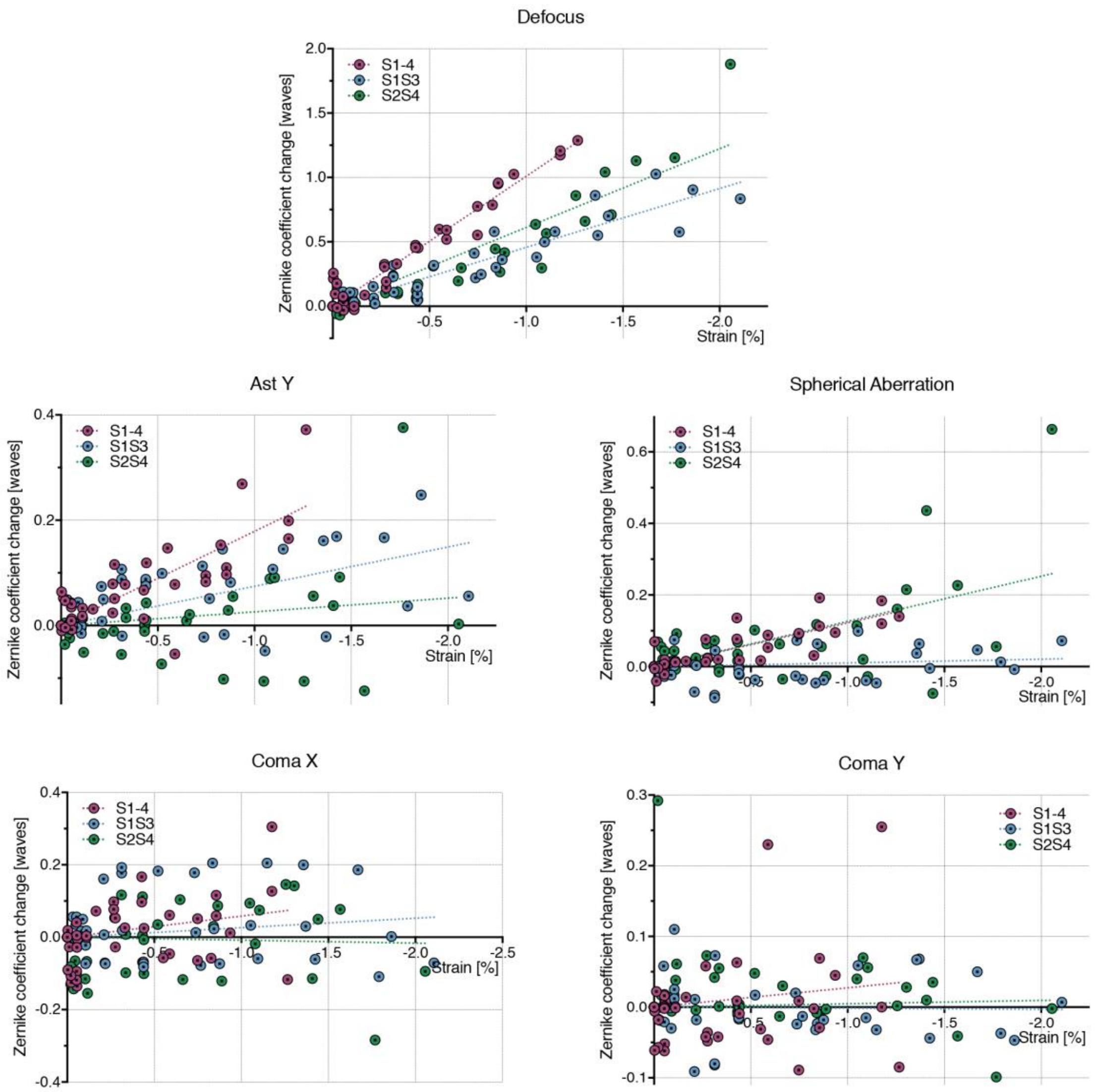

Segments combination	Zernike coefficients	Slope [waves/strain]	R^2	Segments combination	Zernike coefficients	Slope [waves/strain]	R^2	Segments combination	Zernike coefficients	Slope [waves/strain]	R^2
S1-4	Ast X	-0.1018	0.3134	S1S3	Ast X	1.2920	0.9723	S2S4	Ast X	-1.7080	0.9509
	Ast Y	-0.1788	0.6373		Ast Y	-0.0746	0.2203		Ast Y	-0.0260	0.0658
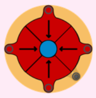	DEFOCUS	-1.0090	0.9482	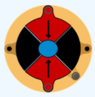	DEFOCUS	-0.4569	0.8672	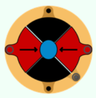	DEFOCUS	-0.6115	0.8525
	SPHA	-0.1221	0.6902		SPHA	-0.0103	0.0417		SPHA	-0.1262	0.3406
	COMA X	-0.0584	0.1114		COMA X	-0.0263	-0.0456		COMA X	0.0082	-0.0418
	COMA Y	-0.0273	0.0443		COMA Y	0.0016	-0.0018		COMA Y	-0.0047	-0.1086

Figure S4. Dependence of the lens aberrations on the electrically induced deformations, for the three driving modes. For each combination of active segments (S1S3, S2S4 and S1-4) and for increasing applied fields (up to 60 V/μm, with 5 V/μm steps), each average Zernike value from the data set in Fig. 3A was matched with the corresponding average strain, at the same field, from the supplementary Fig. S3, to obtain these Zernike-strain plots (which therefore have no error bars). The Ast X-strain plot is presented in Fig. 4 of the paper. Linear fitting lines are added to each plot and the fitting parameters are listed in the tables. R^2 above 0.9 is highlighted in each table; it occurred for defocus with S1-4 and, for horizontal astigmatism, with S1S3 (positive slope) and S2S4 (negative slope).

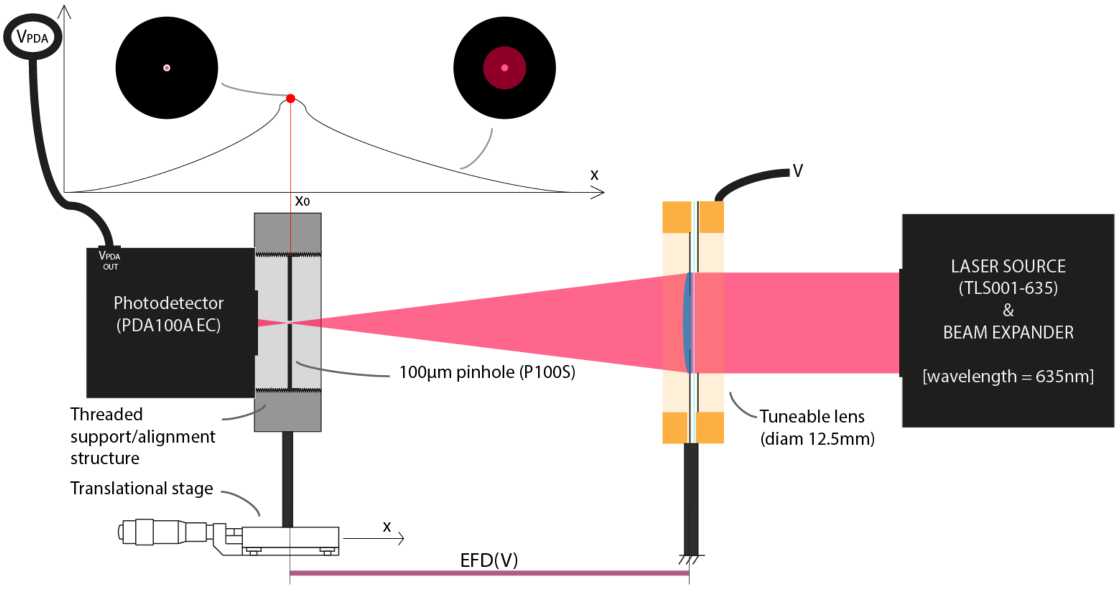

Figure S5. Measurement setup for the effective focal distance (EFD). A collimated and expanded LASER beam is focused by the smart lens while it is driven with radial actuation (S1-4). A photodetector combined with a 100µm-diameter pinhole (aligned with the lens and laser source) are moved parallel to the optical axis (direction *x*) using a translational stage. The photodetector output signal is continuously monitored: at its maximum value, the distance between the pinhole and the lens corresponds to the EFD, as in that condition the focused laser beam goes through the pinhole with minimal reflection and absorption. This measurement was repeated to determine the EFD for different applied electric fields.

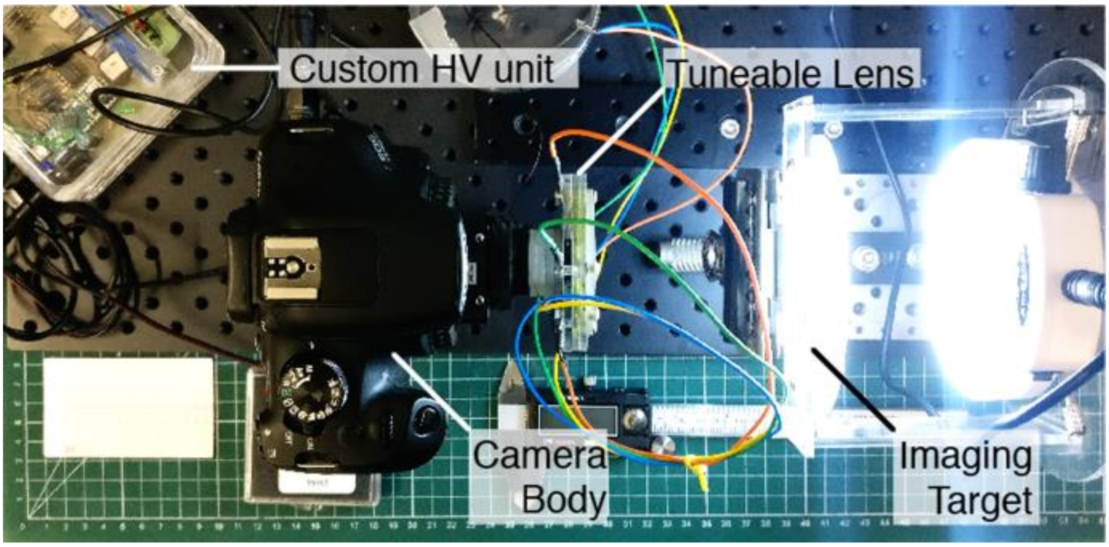

Figure S6. Experimental setup for the imaging tests. The smart lens was mounted on a reflex camera body using a custom adapter and was electrically controlled by an external electrical unit to image various targets while dynamically tuning the astigmatism.
